# Object play in Tsimane children: implications for sex-specific division of labour

**DOI:** 10.1017/ehs.2025.10022

**Published:** 2025-10-23

**Authors:** Ava Moser, Michael D. Gurven, Hillard Kaplan, Benjamin Trumble, Jonathan Stieglitz, Paul Hooper, Daniel Cummings, Adrian Jaeggi, Kathelijne Koops

**Affiliations:** 1 Department of Evolutionary Anthropology, University of Zurich, Zurich, Switzerland; 2 Institute of Evolutionary Medicine, University of Zurich, Zurich, Switzerland; 3 Department of Anthropology, University of California Santa Barbara, Santa Barbara, CA, USA; 4 Economic Science Institute, Chapman University, Orange, CA, USA; 5 School of Human Evolution and Social Change, Center for Evolution and Medicine, Institute of Human Origins, Arizona State University, Tempe AZ, USA; 6 Department of Social and Behavioral Sciences and Institute for Advanced Study in Toulouse, Toulouse School of Economics, Toulouse, France; 7 Department of Anthropology, University of New Mexico, Albuquerque, NM, USA

**Keywords:** object play, tool use, childhood, division of labour, horticulturalists

## Abstract

Sex-specific division of labour and the associated use of different subsistence techniques by males (e.g. hunting) and females (e.g. gathering) has played an important role in shaping human societies. Skills needed in adulthood are practiced in play during childhood and object play has been proposed to foster tool-use skills necessary for adult subsistence techniques. Here, we investigated sex differences in the ontogeny of object play in Tsimane children in Bolivia to understand its potential role in shaping gender-specific adult roles. We used observational data (>80,000 scan samples) from nine Tsimane communities collected between 2002 and 2007. We analysed age and sex differences in general play, object play, and object types. Our results show that both general play and object play peaked in early to middle childhood (3.5–7.5 years of age), with boys spending more time playing. Moreover, boys engaged more with objects related to male-specific roles (e.g. hunting tools), while girls played more with objects related to female-specific roles (e.g. cooking tools). Our findings suggest that object play serves as an adaptive, culturally embedded pathway to develop gender-specific adult skills. Studying developmental patterns of object play across human cultures enriches our understanding of the evolutionary contexts shaping divisions of labour.

## Graphical Abstract

**Figure:**
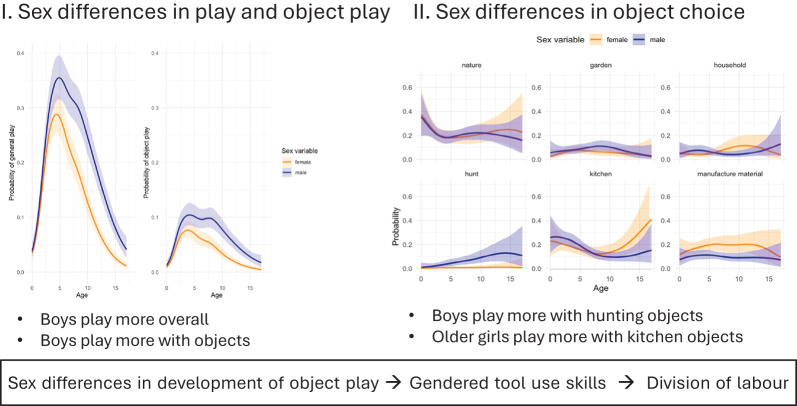

## Social media summary

Sex differences in the ontogeny of object play in Tsimane children reflect gender-specific adult subsistence roles.

## Introduction

Complex technology and pronounced division of labour are defining features of many human societies. The division of labour, particularly along gender lines, may have emerged as an adaptive response to ecological and social demands, enabling individuals to specialize in complementary roles that improved overall efficiency (Gurven & Hill, 2009; Hooper et al., 2015). As a result, the subsistence techniques, and the associated tool-use skills required, differ between men and women in these societies (Kaplan et al., 2009). How, and when, these sex differences in behaviour emerge during development remains to be investigated. Play (with or without objects) is an essential part of development for humans and other animal species (Byers & Walker, 1995; Riede et al., 2018). Play is commonly defined as voluntary and repetitive, offering no immediate or obvious practical benefits, and differs structurally or contextually from serious behaviours, is self-rewarding, and occurs predominantly in relaxed, low-stress states (Burghardt, 2010; Burghardt, 2005). In humans, play has important implications for the physical and cognitive development of children (Edwards et al., 2003). Physical activities like running and climbing enhance motor skills, whereas interactive and pretend play fosters cognitive flexibility and problem-solving skills (Edwards et al., 2003). Object play – defined as manipulating an object in a playful, non-instructional, setting (Wynberg et al., 2022) – may be particularly relevant when considering the development of tool-use skills, as it allows children to explore physical properties of materials, experiment with cause-and-effect relationships, and practise fine motor control (Geary, 1998; Koops et al., 2015a; Smith, 1982). Researchers have therefore hypothesized that object play early in life may foster the development of tool-use skills needed in adulthood (Smith, 1982; Geary, 1998; Koops et al., 2015a, 2015b; Riede et al., 2018). Hence, object play may not only aid in motor skill acquisition and cognitive development but may also prepare children for culturally specific adult subsistence roles shaped by the division of labour.

Patterns of play behaviour often resemble patterns of culturally specific sex differences in adult behaviour (Hewlett, 2017; Lew-Levy et al., 2018). For example, in societies with gendered divisions of labour, play in children has been observed to mirror adult-typical sex-specific behavioural patterns (Lew-Levy et al., 2018). Children from western, educated, industrialized, rich, and democratic (also called ‘WEIRD’; Henrich et al., 2010) societies have been found to display sex differences in the selection of objects during play, preceding the onset of sex-specific play later in childhood (e.g. cars and dolls, Lauer et al., 2015; Servin et al., 1999). Moreover, boys in the United States were found to engage in more object play than girls (Gredlein & Bjorklund, 2005; Pellegrini & Bjorklund, 2004). However, a male-bias in object play is not consistently found across different human cultures (Boyette, 2016; Lew‐Levy et al., 2020). Specifically, no sex difference in object play was reported in Hadza and BaYaka children, and a female-bias was found in Aka and Ngandu children (Boyette, 2016). Hence, investigations of object play are needed across additional human cultures to better understand whether or not sex differences in children’s object play may reflect culturally determined patterns of adult behaviour.

Relatively little is known about the development of general play and object play in children living in non-western societies (but see Lew-Levy et al., 2018; Boyette, 2016; Salali et al., 2019; Fouts et al., 2013). Hunter-gatherer and horticulturalist lifestyles more closely resemble the ecological and social contexts within which humans evolved and share lifestyle factors that distinguish them from western societies. These include reliance on subsistence for food, living in extended family networks within small kin-based communities, relatively high fertility and mortality, and limited access to electronic goods, the internet, and modern medicine (Kaplan et al., 2009). Horticulturalist societies practise uniquely distinct gender-specific divisions of subsistence activities (Kaplan et al., 2009). Moreover, horticulturalists have a different gender division of labour than hunter-gatherers due to the labour demands of horticulture, leading to a shift in the production of carbohydrates from women to men (Kaplan et al., 2009). This shift has resulted in men engaging in physically intensive horticultural labour, while women predominantly perform direct childcare, household tasks and low-strength subsistence work (Gurven et al., 2009; Trumble et al., 2018). Due to the predominant focus on western societies, the role of object play in the development of gender-specific tool-use skills related to the division of labour in non-western subsistence contexts remains largely unexplored.

To investigate the influence of labour division (often referred to as gender roles) on the development of (object) play in children, we focused on the Tsimane, who inhabit the Amazon region of Bolivia. The subsistence practices of the Tsimane centre around slash-and-burn horticulture and cultivating crops (Kraft et al., 2023). The pronounced division of labour between men and women in this horticulturalist society offers the opportunity to examine how sex-specific object play, reflected in the children’s choice of object, may create avenues for children to practise gender-specific roles. We investigated sex differences in the amount of time devoted to general play (i.e. all play), object play (i.e. play with objects), and the types of objects used in play among Tsimane children and adolescents (i.e. below the age of 18 years old). Our study aimed to explore the development of play with three main research objectives: (1) To examine how age influences the engagement in general play and object play, (2) To investigate sex differences in general play and object play development, and (3) To compare object types used in play between male and female children. By examining the development of sex differences in general play and object play within a non-western society, this study aims to clarify how play in children may reflect and reinforce culturally specific gender roles. First (P1), we predict that the frequency with which children engage in general play and object play will peak during early childhood (2–6 years), a period marked by substantial cognitive, social, and physical developments (Bjorklund & Gardiner, 2010; Lewin, 1931; Power, 1999), which are linked to increased play activity and peer interactions (Bjorklund & Gardiner, 2010; Power, 1999). Second (P2), we predict that middle childhood (6–12 years), a crucial stage for gendered socialization, will reveal distinct patterns in the frequency of object play by boys and girls aligned with the division of labour in Tsimane society (Edwards et al., 2003; Lew-Levy et al., 2018). Last (P3), we predict that sex differences in object types used in play reflect adult gendered behaviours and societal roles (Lew-Levy et al., 2018; Riede et al., 2022).

## Methods

### Study population

Tsimane are forager-horticulturalists residing in the lowland Amazonian region of Bolivia across more than 90 villages along rivers and roads (Kraft et al., 2023; Seabright et al., 2023). These villages, which range in size from 50 to 500 individuals, are composed of several multi-generational household clusters (Kraft et al., 2023). Tsimane society revolves around family units, typically monogamous couples (with some sororal polygyny) with an average total fertility rate of nine children (Schniter et al., 2015), often residing close to extended family to form small clusters. Within these household clusters work effort and resource allocation are organized (Kraft et al., 2023; Seabright et al., 2023; Stieglitz et al., 2013). Although semi-sedentary, individuals commonly relocate within and between villages, often for extended stays with relatives (Schniter et al., 2015). Despite the presence of schools in most villages, regular attendance among children was limited during the time of study (Davis & Cashdan, 2019).

Slash-and-burn farming is a key part of Tsimane livelihood, including the cultivation of crops such as plantains and rice (Kraft et al., 2023; Seabright et al., 2023). Men predominantly engage in hunting and wage labour, whereas women take on responsibilities like food processing, childcare, and chicha production. Men and women both actively participate in fishing, fruit collection, and field work, although heavy labour such as chopping trees is limited to men (Kraft et al., 2023). Children as young as 5 years old contribute to family efforts during the harvesting season (Kraft et al., 2023; Stieglitz et al., 2013). Hunting, which involves shotguns, rifles, and bows and arrows, varies from single-day excursions to extended family trips (Kraft et al., 2023). Fishing, a prevalent practice in Tsimane villages, utilizes diverse methods like hook and line, bow and arrow, and nets. Group fishing events incorporate the use of plant poisons, with the catch being distributed among the participants (Kraft et al., 2023; Seabright et al., 2023).

### Ethics statement

Research with the Tsimane was approved by institutional review boards at UC Santa Barbara and at the University of New Mexico. Permissions were obtained from the Gran Consejo Tsimane community leaders and from the study participants.

### Data collection

Data were collected as part of the longitudinal *Tsimane Health and Life History Project*, which was established in 2002 (Gurven et al., 2017), mostly by graduate students at the University of New Mexico and the University of California Santa Barbara (including co-authors J.S. and P.H.). This project spans various topics, including health, growth, development, ageing, economics, and biodemography. In addition, basic medical care is provided to the Tsimane communities. The data set for this study comprises data collected from nine Tsimane communities between 2002 and 2007. To facilitate data collection, households within each community were assigned to clusters, consisting of 2–4 physically adjacent houses. This arrangement allowed researchers to systematically monitor the activities of all residents within these clusters. Household clusters were selected randomly for data collection, and all the clusters were sampled once before sampling the first cluster again (Seabright et al., 2023).

Observational behavioural data were collected in 2- to 3-hour intervals between 0700 h and 1900 h. Within each interval, scan samples of all residents and visitors were conducted every 30 minutes. During scan observations, detailed records were collected of each individual’s location, ongoing activity (or activities if a focal individual was multi-tasking), and interactions with objects in their environment. Play behaviour was coded using an ethogram (Table S2), which included a broad range of play behaviours such as active play, pretend play, and fidgeting (or solo play); furthermore, any activity (e.g. hunting, cooking, etc.) in a play context was classified as such by putting a ‘p’ in front of the respective activity code. For this study, all activities thus coded as ‘play’ were included. If the individual was interacting with an object during a play behaviour, the instance was coded as ‘object play’. The data set used consisted of 27,244 observations of children and adolescents aged 0–18 years old (*N* = 672 participants; Table S4.1 and S4.2). Sex of participants was assigned based on stated sex by the participants or their family members, or by community census, first name, and outward appearance (boys = short hair, pants; girls = long hair, skirts/dresses).

### Object categories

We grouped objects used in play into categories (Table 1) that reflect the Tsimane context and their daily activities, including kitchen, personal, garden, manufacture, hunting, fishing, nature, household, and toy (see Table S1 for definitions).

**Table 1.** **Object categories.** Objects included in the different categories of objects played with by Tsimane children and adolescents

Object categories	Objects included
Kitchen	Pan, bowl, can, strainer, pot, meat grinder, spoon, cup, knife, metal mug, glass, plate, metal strainer, gallon, dishes, tacu (for rice pounding), bat (pound rice/hit food), shell, erepaj (traditional bowl), weigh scale
Personal	Hair tie, condom, toothbrush, comb, tobacco
Garden	Shovel, rope, water container, chainsaw, wheelbarrow
Manufacture	Non-jatata leaves, bead, bag, mat, pole, loom, cotton, box, shuru’ (bamboo), walls, woven item, basket, needle, bookbag, panel, nail, wire, hammer, tool, yarn
Hunting	Slingshot, arrow, bow, trap, axe, machete, rifle, shotgun, shotgun shell, bullet
Fishing	Net, line, hook, chito and washi (fish poison)
Nature	Stick, rock, tree, grass, piece of dirt, flower, plant, bee, termite mound
Household	Pencil sharpener, broom, radio, cassette, ladder, blanket, umbrella, balloon, whistle, flashlight, syringe, scissors, plastic, pen, bin, string, bucket, battery, mosquito net, sewing machine, lighter, soap, writing material, plastic bag
Toy	Toy, toy truck, toy boat, toy gun, book, toy house, soccer ball, swing, doll, ball, stuffed toy, volleyball, toy whip

### Play data

The data set showed a balanced distribution of individuals across the binary sex variable with 325 female and 347 male children and adolescents. The distribution of age showed approximately 60 children per year up to the age of 10 years old. Beyond this age, there was a decrease to 30–40 children per year included in this study. This decline may be attributed to factors such as older children and adolescents attending school or beginning to involve themselves in adult-like responsibilities (e.g. hunting, gardening), resulting in less frequent presence near the home environment where most observations were conducted.

### Data analyses

Bayesian regression models were implemented with the brms package (Bürkner, 2017) in R (version 4.3.2). Age was modelled with splines to capture nonlinear developmental patterns in play behaviour. Personal ID was included as a random effect in all models to quantify individual differences and partition within versus between individual variances (Table 2). We used weakly regularizing priors (intercept = normal(0, 2); standard deviation of random effects = exponential(1) in Bernoulli models and exponential(2) priors in categorical models). These priors help the model converge faster by constraining the likely parameter space, without imposing overly strong conservatism.

**Table 2.** Summary of models 2a (general play) and 2b (object play). All parameters are on the logit scale, and posterior distributions of parameter estimates are summarized by their mean, standard error, and 95% credible intervals. The smooth term (Sds(Age_1)) reflects the variability in age-related trends. The group-level effect (Sd(Intercept PID)) accounts for individual differences in baseline play levels. Population-level effects include main effects of age and sex and their interaction, including the non-linear spline terms nsAge31, nsAge32, nsAge33

	Model 2a (General play)				Model 2b (Object play)			
Parameter	Mean	SE	Lwr 95%	Upr 95%	Mean	SE	Lwr 95%	Upr 95%
**Smooth Terms**								
Sds(Age_1)	1.93	0.69	0.88	3.57	2.69	0.85	1.26	4.59
**Group-Level effects**								
Sd (Intercept PID)	0.61	0.03	0.56	0.68	0.66	0.04	0.57	0.74
Intercept	−2.74	0.7	−4.11	−1.32	−4.1	0.75	−5.59	−2.65
Sex = male	0.12	0.21	−0.30	0.54	0.33	0.28	−0.22	0.88
nsAge31	−0.6	1.26	−3.18	1.8	−1.29	1.43	−4.20	1.42
nsAge32	1.83	1.46	−1.09	4.68	2.18	1.56	−0.84	5.28
nsAge33	−1.33	1.61	−4.38	1.89	−0.4	1.75	−3.73	3.09
Sex = male:nsAge31	0.71	0.27	0.17	1.25	0.65	0.36	−0.04	1.34
Sex = male:nsAge32	1.12	0.52	0.12	2.16	0.81	0.69	−0.55	2.16
Sex = male:nsAge33	1.17	0.31	0.56	1.77	1.06	0.43	0.24	1.90
sAge_1	0.45	1.75	−3.04	3.81	1.11	1.81	−2.40	4.62

To model the development of general play (all instances of play) and object play across age, we used two Bernoulli models with age splines (models 1a and 1b). In these Bernoulli models, every behavioural observation constituted a ‘trial’, with play observations coded as ‘successes’ (1) and all other observations as ‘failures’ (0). To test for sex differences in the frequency of general play and object play, we added sex and the interaction between sex and age to the models (models 2a and 2b). Finally, to test for the effect of age and sex on object types used in object play, we used a multinomial model with object category as the response variable, and an age spline with sex and the interaction of age and sex as the explanatory variables (model 3).

Bayesian models estimate a posterior distribution for all parameters. We here mostly summarize these posteriors using their means, the standard errors of the mean, and 95% credible intervals, but readers should note that these are essentially arbitrary choices. More intuitively, the degree of confidence in a given association can be computed as the proportion of the posterior distribution that is greater or smaller than 0, hence we also present these posterior probabilities where appropriate.

## Results

**Figure 1.**
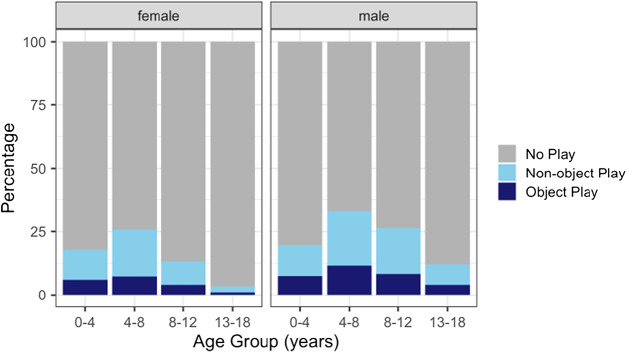
**Time dedicated to play.** Proportion of time dedicated to object play versus non-object play activities in female and male children across different age groups.

Of 5,471 (girls: 2,143, boys: 3,328) observations of general play, a total of 1,827 (girls: 661, boys: 1,166) observations were categorized as object play. Raw data summary plots show that boys dedicated a larger proportion of their time to general play activities across all age groups, and that the frequency of non-object play and object play is highest between the ages of 4 and 8 years old in both boys and girls (Fig. 1).

### Development of general play and object play

**Figure 2.**
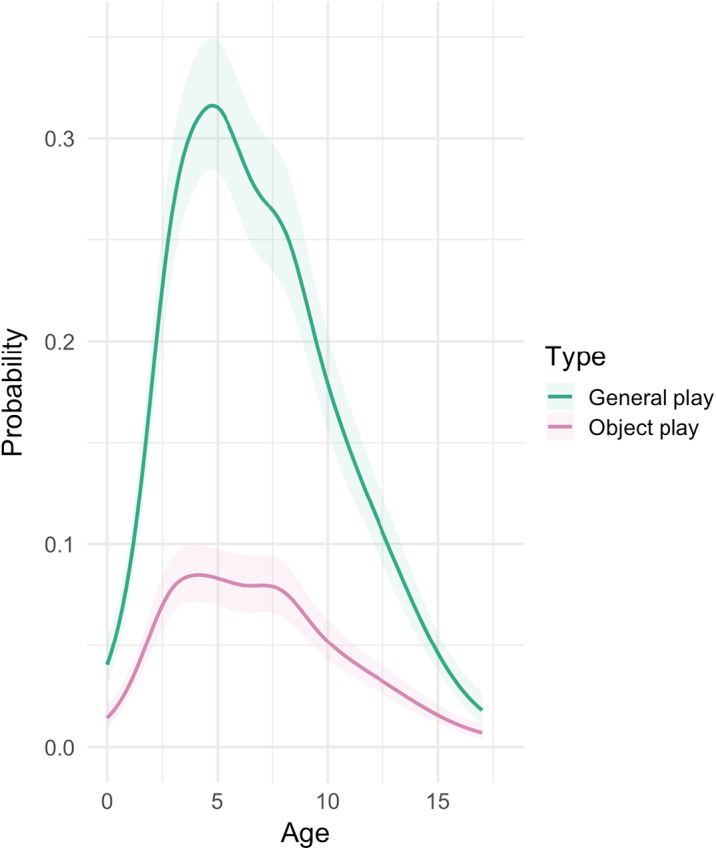
**Probabilities of general play and object play.** Bayesian analysis of general play and object play probabilities as a function of age (in years). The lines show the predicted probabilities across age and the shaded areas represent the 95% credible intervals around those predictions.

Models 1a and 1b (Table S3) revealed distinct age-related play patterns in childhood, characterized by a sharp increase and slower decline with age (Fig. 2). The likelihood of general play activities reached its maximum at approximately 5 years of age, with an estimated peak probability of around 30% (Fig. 2), i.e. children spent almost a third of their time playing. The probability of engagement in general play dropped progressively beyond this age. A parallel trajectory was observed for object play (Fig. 2). Notably, the peak for object play occurred within the same age range, but settled at a lower peak probability of around 8%. A plateau of object play was observed from around 3.5 to 7.5 years old, indicating sustained engagement in object play activities during this developmental period.

### Sex differences in play

**Figure 3.**
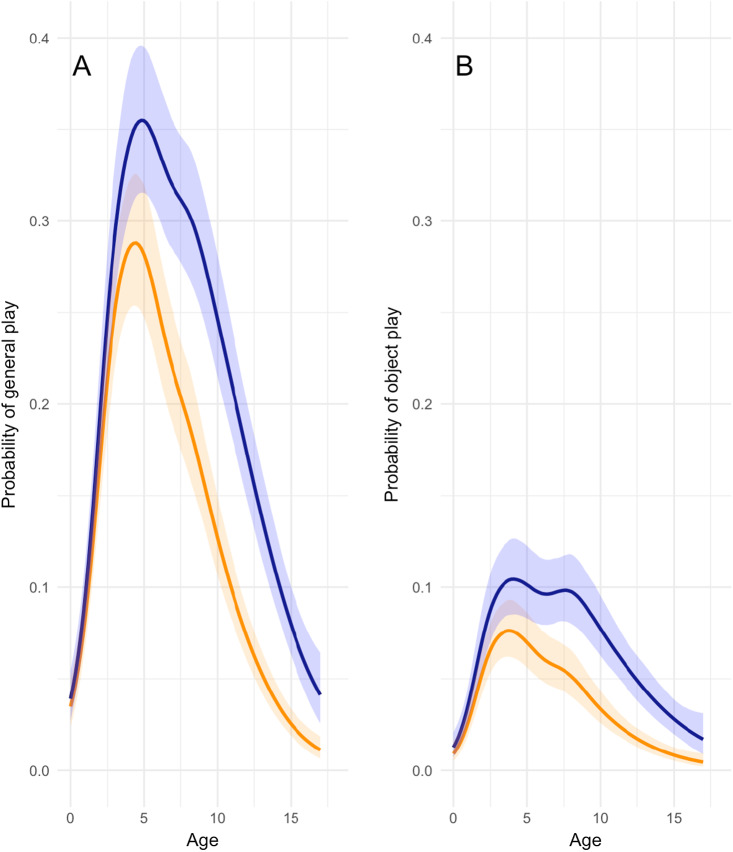
**Sex differences in probability of general play (A) and object play (B).** Bayesian analyses of general play (A) and object play (B) probability, considering the interaction of age and sex. The lines show the predicted probabilities across age for each sex, and the shaded areas represent the 95% credible intervals around those predictions.

Models 2a and 2b suggested a positive effect of male sex on the probabilities of both general play (mean slope = 0.12, standard error = 0.21, 95% credible interval = [−0.3, 0.54], Table 2) and object play (mean slope = 0.33, standard error = 0.28, 95% credible interval = [−0.22, 0.88], Table 2). While the credible intervals for both effects included zero, the posterior distributions indicated a 70% probability that the effect of male sex on general play was positive, and an 88% probability for object play. These results suggest modest, but uncertain, sex differences in the likelihood of engaging in general play and object play. However, the interaction between sex and age was more strongly supported (Fig. 3), with most credible intervals for the age-spline sex interaction terms not containing zero (Table 2).

The probability of general play did not differ between the sexes at very young ages (approximately below 2.5 years old), contributing to the high uncertainty in the overall effect of male sex (Fig. 3A). However, boys exhibit a higher peak probability of engaging in general play (ca. 35% probability) around 5 years of age (Fig. 3A). After 5 years of age, the probability begins to gradually decrease. Conversely, girls demonstrate a similar peak albeit at a slightly lower probability (ca. 29%). Sex-specific patterns in the development of object play were also found (Fig. 3B). The probability for object play in boys shows a peak at around 5 years old, akin to the general play pattern, but at a lower probability (ca. 12.5%). The persistence of object play is evident in a plateau extending beyond the peak age, up until around 7.5 years old. Girls follow a parallel trend, with a lower peak probability (ca. 8%) than that of boys and a more pronounced decrease post-peak (Fig. 3B).

### Sex differences in object type choice

**Figure 4.**
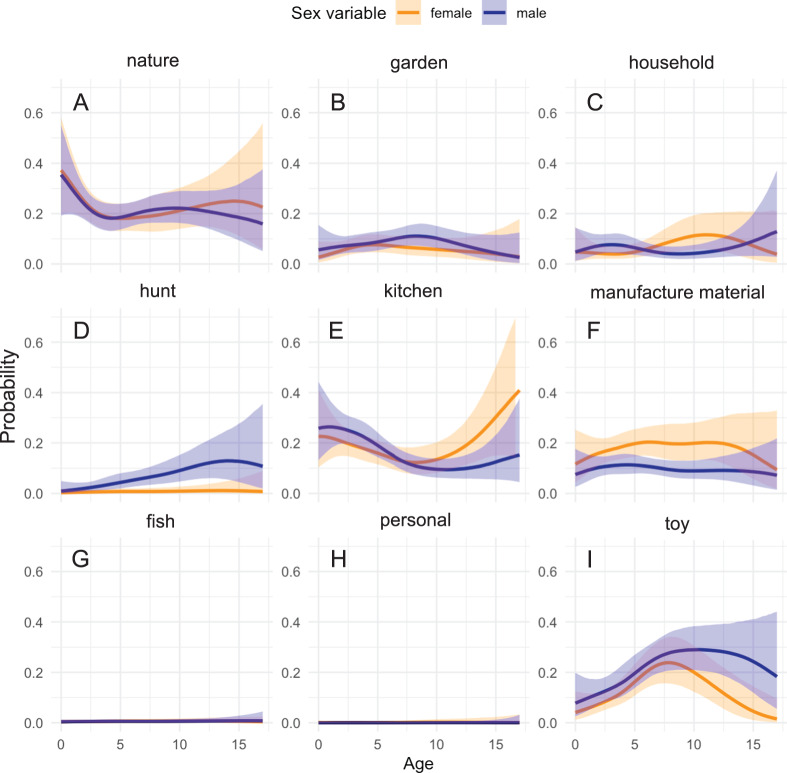
**Sex differences in probability of playing with different object types.** Probability of Tsimane children playing with objects of different categories: nature (A), garden (B), household (C), hunt (D), kitchen (E), manufacture material (F), fish (G), personal (H), toy (I). The lines represent the estimated probabilities for males (blue) and females (orange) across age groups, highlighting the interaction between sex and age on object type preferences.

Model 3 analysed how age and sex influence preferences for different types of play objects. The model suggested a marginal positive main effect of male sex on the probabilities for the ‘hunt’ category (estimate = 1.11, standard error = 0.92, 95% credible interval = [−0.72, 2.9], posterior probability = 88.6%), and the ‘garden’ category (estimate = 0.70, standard error = 0.81, 95% credible interval = [−0.88, 2.26], posterior probability = 80.7%). A negative effect was suggested on ‘manufacture’ (estimate = − 0.46, standard error = 0.72, 95% credible interval = [−1.88, 0.95], posterior probability = 74.2%), and virtually no main effect of sex for the ‘kitchen’ (estimate = 0.11, standard error = 0.69, 95% credible interval = [−1.22, 1.47], posterior probability = 56.7%), ‘household’ (estimate = − 0.02, standard error = 0.81, 95% credible interval = [−1.60, 1.58], posterior probability = 52.2%) and ‘nature’ (estimate = − 0.07, standard error = 0.69, 95% credible interval = [−1.42, 1.28], posterior probability = 54.4%) categories. Age and sex interactions are best examined visually (Fig. 4). We did not examine the ‘fish’ and ‘personal’ categories in detail due to their low frequency (Fig. 4) in the data set, and we excluded the ‘toys’ category from further analysis as it was not central to our research focus and encompassed a broad range of object types.

Despite the model initially suggesting a positive effect for males in the ‘kitchen’ category, visualizations reveal a shift in this trend post-middle childhood, when females increasingly engage with kitchen-related objects (Fig. 4E). Specifically, after the age of 7 years old, there is a notable divergence: the likelihood of girls interacting with kitchen objects rises sharply, doubling from 20% to nearly 40%, in contrast to boys, whose likelihood of engagement remains at around 20%. This finding suggests a pivotal developmental period influencing the play behaviour of female Tsimane children in relation to kitchen objects. In contrast, when considering hunting-related objects (Fig. 4D), girls consistently showed negligible engagement, while boys demonstrated a steady increase in engagement, with a notable climb to over 10% probability as they age. This gradual but persistent increase implies a growing interest in hunting-related objects among male Tsimane children as they grow older. The analysis of objects related to manufacturing activities (e.g. weaving, canoe building) and manufacture material objects (e.g. cotton, nail, wool, hammer) further substantiated sex-specific trends. Throughout childhood, girls consistently showed a higher probability – about 20% – of engagement with manufacture objects compared to boys, who hovered around the 10% mark. For the garden (Fig. 4B), nature (Fig. 4A), and household (Fig. 4C) objects, no sex differences were observed. Engagement with garden-related objects shows a subtle increase with age yet remained under a 20% probability for both sexes. Play with natural objects started with a slightly higher probability in early childhood but exhibited a general decline as children got older (Fig. 4A). Finally, object play with general household objects maintained a consistent probability across ages (around 10%), suggesting a similar involvement in general activities in the home for both boys and girls (Fig. 4C).

## Discussion

We examined sex differences in both general play behaviour and object play in Tsimane children in Bolivia. We found that Tsimane children allocated on average a quarter of their time to general play (Fig. 1), which is similar to proportions reported for forager societies, like the Hadza in Tanzania and the BaYaka in the Republic of Congo (Lew‐Levy et al., 2020). The significant proportion of time spent playing across cultures supports the idea that general play is an essential part of childhood (Byers & Walker, 1995; Riede et al., 2018). Moreover, we found sex-specific developmental patterns that reflect the culturally specific gendered division of labour in Tsimane society. Notably, the results revealed that boys, particularly in middle and late childhood, tend to spend more time playing in general, as well as more time playing with objects compared to girls. This finding suggests an early differentiation in the allocation of time to different activities between boys and girls.

Moreover, the object types played with showed sex-specific tendencies that align with the division of labour observed in adulthood. Specifically, older girls played more with objects commonly used in the kitchen, such as pans, bowls, and knives, whereas boys showed a higher probability to engage with objects used in the hunting context such as slingshots, arrows, bows, and guns. These findings suggest that object play may serve as an avenue for practising tool-related skills that are relevant in adult subsistence roles, such as food processing or hunting. The observed sex differences in the developmental patterns of play behaviour provide insights into the formative influence of play on the acquisition of culturally relevant skills (see also Stieglitz et al., 2013). Overall, these findings not only underscore the significant role of sex in the developmental trajectory of object play but also suggest a complex interplay between age, sex, and societal norms that may influence children’s play behaviour with objects. Our data do not provide information on whether children are choosing to engage with the different types of objects, or whether caregivers encourage or enforce gendered play. However, most research on social learning in subsistence societies emphasizes high autonomy and limited teaching (Boyette & Hewlett, 2018; Hewlett et al., 2011), suggesting independent choice of children to engage with objects in play. Further research using focal follows and ethnographic interviews could address in more detail the potential role of parents, or other group members, in encouraging children to engage with different object types.

### Development of play

We found that general play and object play in Tsimane children peaked in early to middle childhood (3.5–7.5 years old), in line with observations in forager cultures such as the Hadza and the BaYaka (Lew‐Levy et al., 2020). This finding supports our first prediction (P1) that early childhood represents a critical phase for engaging in play, particularly object play, as it is instrumental for cognitive and physical development (Feinstein & Bynner, 2004). The noticeable plateau in the probability of object play, but not general play, during these years suggests an important extended period of increased object-interest, potentially linked to the acquisition of object-related skills (Riede et al., 2018). Thus, the peak of play activities during this crucial developmental period further affirms the critical role of play in the developmental trajectory of children.

### Sex differences in play development

We observed pronounced sex differences in play patterns of Tsimane children emerge with age from about 4 years old, with boys showing significantly higher levels of engagement in both general and object play activities. The observed sex differences in play behaviours among Tsimane children resonate with the broader discourse on gendered socialization through play (Edwards et al., 2003; Lew-Levy et al., 2018), where play activities are intertwined with the acquisition of gender-specific adult competencies. The existing literature on sex differences in object play across cultures shows conflicting findings. Lew-Levy and colleagues (2020) observed no significant sex differences in object play in Hadza and BaYaka children. In the Aka and Ngandu, girls were found to engage more in play involving objects than boys (Boyette, 2016). And in a study with American children, boys were found to be more inclined towards object play (Gredlein & Bjorklund, 2005). Hence, significant cross-cultural variation exists in observed sex differences (or absence thereof) in object play.

Our findings showed, as predicted (P2), that sex differences in general play and object play become pronounced in middle childhood (4–8 years), known as a pivotal time for the acquisition of gender norms (Konner, 2010). While mixed-sex play is common during the early years, as children grow older, they tend to imitate the behaviours of adults of their own sex, leading to more gender-specific behaviours (Lew-Levy et al., 2018). Children in more sedentary societies experience different learning environments than children in more nomadic societies, with girls generally spending more time near the house and thus transitioning earlier to (non-play) activities considered part of their culturally specific gender role (Draper, 1975; Draper & Cashdan, 1988). This could be an explanation for the strong disparities at the age of 7 years old when girls engage almost 50% less in object play compared to boys (i.e. males 10%, females 5%). Overall, our findings suggest that both general play and object play may serve as precursors to the gendered division of labour later in life in Tsimane children. Such sex-differentiated play, akin to patterns of social play and object play in non-human primates and other animals (Koops et al., 2015b; LaFreniere, 2011; Marley et al., 2022), suggests a shared evolutionary basis for developmental differences in play foreshadowing sex differences in adult behaviour.

### Sex differences in object choice

Our findings also indicated that boys and girls seem to play with different object types, which aligned with gender differences in adult roles. Boys engaged with hunting-related objects more frequently whereas older girls favoured kitchen-related objects. These results are in line with the hypothesis that object play early in life catalyses the development of tool-use skills needed later in life (Koops et al., 2015a, 2015b; Riede et al., 2018). Boys engaging more frequently in play that involves objects related to hunting may indicate preparation for skills associated with men in Tsimane culture (Kraft et al., 2023). Our findings align with the cross-cultural findings from 54 hunter-gatherer societies which reported that boys are more likely to use risky objects, such as machetes, and guns (Lew-Levy et al., 2022). In contrast, the growing preference of girls for kitchen-related objects in play from about 7 years of age suggests that older girls learn about subsistence skills of women in Tsimane society. These findings align with previous research showing that girls tend to stay near the home more so than boys in settled societies, which leads to a more pronounced sexual division of labour (Draper, 1975; Draper & Cashdan, 1988).

Additionally, the observed (female-biased) sex difference in play with manufacturing materials, seems to contradict the hypothesis (and associated prediction P3) that object play serves as preparation for the division of labour, as both men and women in Tsimane society are involved in manufacturing activities. However, this discrepancy might be explained by the methodology of our study, which focused on direct observations. The manufacturing activities predominantly involving men, such as canoe building, often occur outside of the observed village setting. In contrast, the majority of manufacturing done by women, such as weaving traditional cotton bags or reed mats, occurs mostly at home, possibly leading to an overrepresentation in the play observed with these objects. The observed sex differences in play with kitchen and hunting objects (albeit with statistical uncertainty) support the hypothesis that play serves as a precursor to the acquisition of adult competencies, including subsistence tool use, and mirrors the gender divisions of labour observed in Tsimane society (Bock & Johnson, 2004; Kraft et al., 2023). In sum, the sex differences in play observed in this study provide evidence of gender role socialization in a non-western context and the possible role of object play in the acquisition of gender norms during development.

### Future directions

Future research focused on the development of play and object play in the Tsimane should include targeted data collection, and the ethogram for object play should extend beyond identifying the objects used in play to also include the types of interaction with these objects, thereby allowing for a more nuanced classification of sex-specific behaviours. Moreover, in this study, we used data collected during scan sampling within a village setting only. Hence, this approach excludes play activities occurring outside the predefined village observation area. Future research employing focal follows of children could provide additional insights into the development of object play in Tsimane children by including activities beyond the immediate household setting.

### Conclusions

Our findings on general play and object play among Tsimane children underscore the evolutionary and developmental significance of play as a way of acquiring tool use skills and culturally specific gender roles. In line with evolutionary theories, the observed sex differences in play behaviours suggest that object play serves as an adaptive mechanism, preparing children for future roles within their society. Male children’s engagement with hunting-related objects and female children’s preference for kitchen-related items reflect an early orientation towards adult responsibilities, supporting the notion that play provides a structured setting for the practice of culturally relevant skills. This study highlights the complex interplay between play, cognitive development, and learning, illustrating how early-life behaviours can prepare for adult divisions of labour. By examining these patterns within a non-western society, we extend our understanding of cross-cultural variability of play. Future cross-cultural research on object play is crucial for our understanding of how play shapes developmental trajectories across human societies and provides insights into the role of play in preparing children for adult roles in their specific cultural setting.

## Supporting information

Moser et al. supplementary material Moser et al. supplementary material

## Supplementary material

The supplementary material for this article can be found at https://doi.org/10.1017/ehs.2025.10022.

## Research Transparency and Reproducibility

Individual-level data are stored in the Tsimane Health and Life History Project (THLHP) Data Repository and are available through restricted access for ethical reasons. THLHP’s highest priority is the safeguarding of human subjects and minimization of risk to study participants. The THLHP adheres to the ‘CARE Principles for Indigenous Data Governance’ (Collective Benefit, Authority to Control, Responsibility, and Ethics), which assure that the Tsimane (1) have sovereignty over how data are shared, (2) are the primary gatekeepers determining ethical use, (3) are actively engaged in the data generation, and (4) derive benefit from data generated and shared for use whenever possible. The THLHP is also committed to the ‘FAIR Guiding Principles for scientific data management and stewardship’ (Findable, Accessible, Interoperable, Reusable). Requests for individual-level data should take the form of an application that details the exact uses of the data and the research questions to be addressed, procedures that will be employed for data security and individual privacy, potential benefits to the study communities, and procedures for assessing and minimizing stigmatizing interpretations of the research results (see the following webpage for links to the data sharing policy and data request forms: https://tsimane.anth.ucsb.edu/data.html). Requests for individual-level data will require institutional IRB approval (even if exempt) and will be reviewed by an Advisory Council composed of Tsimane community leaders, community members, Bolivian scientists, and the THLHP leadership. The study authors and the THLHP leadership are committed to open science and are available to assist interested investigators in preparing data access requests. All R-code used in our analyses can be found here: https://github.com/adrianjaeggi/TsimaneObjectPlay.
