# Supplementary material for: Object play in Tsimane children: implications for sex-specific division of labour
Source: Evol Hum Sci. 2025 Oct 23;7:e37. doi: 10.1017/ehs.2025.10022 (PMC12645321; doi:10.1017/ehs.2025.10022)
Supplement: Moser et al. supplementary material [file S2513843X25100224sup001.docx]

# **Supplementary materials**

**Table S1.** Summary of play activity categories, their associated definitions, and the gender most expected to perform the related adult work. The table outlines specific play types such as kitchen or hunting activities, indicative of traditional gender roles, and general play categories, illustrating the societal expectations reflected in children's play behaviour.

| **Category** | **Definition** | **Sex** |
| --- | --- | --- |
| Kitchen | Play activities related to cooking or kitchen chores | Female |
| Manufacture | Play involving building or creating objects | Both |
| Hunt | Play activities related to hunting | Male |
| Garden | Play activities related to garden work | Both |
| Fish | Play activities related to fishing | Both |
| Childcare | Role-playing scenarios related to taking care of a baby or doll | Female |
| Social | Play focused on social interactions and relationships | Both |
| Household | Pretend chores and tasks typically done at home | Female |
| Active play | Physical and energetic play activities | Both |
| Play general | General, all-encompassing play without a specific focus | Both |
| Normal things | Everyday activities mimicked in play | Both |
| Fidget | Play involving fidgeting or repetitive movements | Both |

**Table S2.** Ethogram for play behaviours with the associated code and the description of the different behaviours.

| **Behaviour** | **Code** | **Description** |
| --- | --- | --- |
| fidget | PGFG | to manually manipulate an object passively with a low |
|  |  | level of concentration and energy expenditure |
|  |  | (adults and children both) |
|  |  |  |
| **Play Codes** |  |  |
| *any activity may become a play activity, just add "P" to the existing code and fill out all the sections of the database* | | |
| *pertaining to play with the following codes:* |  |  |
| play | PLY | most general code, use more detail wherever possible |
| play with others | PLYO | a general code, only use when no other play codes apply |
| play with self | PLYS | a general code, only use when no other play codes apply |
| play "house" | PHOUSE |  |
| play chase | PCHASE |  |
| play soccer | PSOC |  |
| play, manufacture | PLMU |  |
| play, balance | PGBL | children play by walking, balancing, on a branch |
|  |  |  |
| *be careful to distinguish play from actual engagement in activity, for example, if a child is pretending to make chicha* | | |
| *with dirt, that is play, but if the child is helping her mother make chicha, her activity would not be considered play* | | |
| *hunting: if small children are hunting lizards, other non-game-prey, practicing to shoot the bow and arrow, code as play* | | |
| *if children leave the cluster, enter the woods, appear to be in pursuit of actual game, code as hunting, and record in-out* | | |
| *times wherever possible fishing: if children leave the cluster to fish, or produce fish, code as fishing, and record in-out* | | |
| *times and production details on the resource production data sheet* | |  |
|  |  |  |

**Table S3.** Summary of models 1a (general play) and 1b (object play). All parameters are on the logit scale, and posterior distributions of parameter estimates are summarised by their mean, standard error and 95% credible intervals. The smooth term (Sds(Age_1)) reflects the variability in age-related trends. The group-level effect (Sd(Intercept PID)) accounts for individual differences in baseline play levels. Population-level effects include main effects of age.

|  | **Model 1a (General play)** | | | | **Model 1b (Object play)** | | | |
| --- | --- | --- | --- | --- | --- | --- | --- | --- |
| **Parameter** | **Mean** | **SE** | **Lwr 95%** | **Upr 95%** | **Mean** | **SE** | **Lwr 95%** | **Upr 95%** |
| **Smooth Terms**  Sds(Age_1) | 2.43 | 0.68 | 1.39 | 4.06 | 2.37 | 0.73 | 1.22 | 4.09 |
| **Group-Level effects**  Sd (Intercept PID) | 0.70 | 0.03 | 0.64 | 0.77 | 0.74 | 0.05 | 0.65 | 0.83 |
| Intercept  sAge_1 | -1.68  3.02 | 0.04  1.37 | -1.75  0.31 | -1.61  5.69 | -3.02  3.53 | 0.05  1.50 | -3.12  0.56 | -2.93  6.51 |

**Table S4.1.** Number of observations per age year for the overall data set (N = 672 participants).

| **Observation per age year** | |
| --- | --- |
| 0 | 2574 |
| 1 | 1829 |
| 2 | 2204 |
| 3 | 2102 |
| 4 | 1873 |
| 5 | 1865 |
| 6 | 1844 |
| 7 | 1743 |
| 8 | 1513 |
| 9 | 1288 |
| 10 | 1735 |
| 11 | 940 |
| 12 | 1302 |
| 13 | 1154 |
| 14 | 832 |
| 15 | 805 |
| 16 | 955 |
| 17 | 684 |
| **Male proportion** | |
| 0.505 | |
| **Average obs per PID** | |
| 32.37 | |

**Table S4.2.** Number of observations per age year for the ‘general play’ data set (N = 672 participants).

| **Observation per age year** | |
| --- | --- |
| 0 | 150 |
| 1 | 269 |
| 2 | 507 |
| 3 | 639 |
| 4 | 539 |
| 5 | 600 |
| 6 | 550 |
| 7 | 509 |
| 8 | 407 |
| 9 | 293 |
| 10 | 356 |
| 11 | 177 |
| 12 | 166 |
| 13 | 143 |
| 14 | 69 |
| 15 | 36 |
| 16 | 45 |
| 17 | 16 |
| **Male proportion** | |
| 0.608 | |
| **Average obs per PID** | |
| 8.34 | |
